# Leaching of copper and nickel in soil-water systems contaminated by bauxite residue (red mud) from Ajka, Hungary: the importance of soil organic matter

**DOI:** 10.1007/s11356-015-4282-4

**Published:** 2015-03-12

**Authors:** Cindy L. Lockwood, Douglas I. Stewart, Robert J. G. Mortimer, William M. Mayes, Adam P. Jarvis, Katalin Gruiz, Ian T. Burke

**Affiliations:** 1 School of Earth and Environment, University of Leeds, Leeds, LS2 9JT UK; 2 School of Civil Engineering, University of Leeds, Leeds, LS2 9JT UK; 3 Centre for Environmental and Marine Sciences, University of Hull, Scarborough, YO11 3AZ UK; 4 School of Civil Engineering and Geosciences, Devonshire Building, Newcastle University, Newcastle Upon Tyne, NE1 7RU UK; 5 Department of Applied Biotechnology and Food Science, Budapest University of Technology and Economics, St Gellért sq. 4, 1111 Budapest, Hungary; 6 Present Address: Centre for Applied Geoscience, Eberhard-Karls-University Tübingen, 72076 Tübingen, Germany; 7 Present Address: School of Animal, Rural and Environmental Sciences, Nottingham Trent University, Brackenhurst Campus, Southwell, Nottinghamshire NG25 0QF UK

**Keywords:** Kolontár, Ajka, Red mud, Nickel, Copper, DOC, Soil organic matter, Solid phase extraction

## Abstract

Red mud is a highly alkaline (pH >12) waste product from bauxite ore processing. The red mud spill at Ajka, Hungary, in 2010 released 1 million m^3^ of caustic red mud into the surrounding area with devastating results. Aerobic and anaerobic batch experiments and solid phase extraction techniques were used to assess the impact of red mud addition on the mobility of Cu and Ni in soils from near the Ajka spill site. Red mud addition increases aqueous dissolved organic carbon (DOC) concentrations due to soil alkalisation, and this led to increased mobility of Cu and Ni complexed to organic matter. With Ajka soils, more Cu was mobilised by contact with red mud than Ni, despite a higher overall Ni concentration in the solid phase. This is most probably because Cu has a higher affinity to form complexes with organic matter than Ni. In aerobic experiments, contact with the atmosphere reduced soil pH via carbonation reactions, and this reduced organic matter dissolution and thereby lowered Cu/Ni mobility. These data show that the mixing of red mud into organic rich soils is an area of concern, as there is a potential to mobilise Cu and Ni as organically bound complexes, via soil alkalisation. This could be especially problematic in locations where anaerobic conditions can prevail, such as wetland areas contaminated by the spill.

**Electronic supplementary material:**

The online version of this article (doi:10.1007/s11356-015-4282-4) contains supplementary material, which is available to authorized users.

## Introduction

The accidental release of ~1 million m^3^ (Reeves et al. 2011; Adam et al. 2011) of bauxite processing residue (red mud) from the Ajkai Timfoldgyar Zrt alumina plant in Ajka, western Hungary, in October 2010 focused world attention on the public health and environmental hazards associated with red mud. The spill caused damage to property, serious injuries and killed 10 people (Enserink 2010; Adam et al. 2011). An estimated 40 km^2^ of low lying agricultural land and riparian wetlands were affected and the red mud was transported 120 km downstream by rivers, eventually reaching the Danube (Reeves et al. 2011; Mayes et al. 2011).

Red mud is the name given to the fine fraction residue produced during alumina extraction from bauxite by the Bayer process. The composition of red mud is dependent upon the bauxite ore used (Hind et al. 1999; Liu et al. 2007) but is typically comprised of iron oxides, quartz, sodium aluminosilicates, titanium dioxide, calcium carbonate/aluminate and sodium hydroxide (Hind et al. 1999; Grafe et al. 2011; Gelencser et al. 2011). Red mud also contains elevated concentrations of potentially toxic metal(loid)s including Al, As, Cr, Cu, Ni, Mo, V and Zn. Although the majority of these problematic elements are found associated with sparingly soluble minerals (Mayes et al. 2011; Rubinos and Barral 2013), several oxyanion-forming elements (e.g. Al, As, Mo and V) have been found to be mobile in waters associated with the red mud (Lockwood et al. 2014; Lehoux et al. 2013; Burke et al. 2013; Milacic et al. 2012). The use of NaOH during the Bayer process means that red mud is very caustic unless the waste is neutralised prior to disposal (Grafe et al. 2011; Power et al. 2011). At Ajka, the red mud had a pH >12 (Adam et al. 2011), making it a hazardous substance as defined by the Basel Convention (Secretariat of the Basel Convention 2011). It is also highly saline and sodic (e.g. Ajka leachate had conductivity of up to 160 mS cm^−2^); therefore, Na stress to soils and plants has been noted as a consequence of the Ajka spill (Ruyters et al. 2011).

Several studies at Ajka have investigated the effects of red mud on human health (Gelencser et al. 2011), soil toxicity (Anton et al. 2012), freshwater and soil ecology (Klebercz et al. 2012; Rekasi et al. 2013), the mobility of red mud associated trace metals in the wider environment (Mayes et al. 2011; Burke et al. 2012; Lehoux et al. 2013), and the effects dosing of rivers and streams with acid and gypsum to reduce pH (Renforth et al. 2012; Burke et al. 2013). After the Ajka disaster, the affected land was treated in two different ways: (1) removal of the red mud where deposits were >5 cm and (2) ploughing the red mud into land where deposits were <5 cm. The land clean-up began a few weeks after the disaster, but in some areas, the red mud covered the soils for several months (Rekasi et al. 2013), and remediation of wetland areas was not attempted.

Humic acids are an important component of soil organic matter (SOM) whose solubility varies with pH. They are produced by microbial degradation of plant and animal residues and represent a range of chemically similar compounds that contain carboxyl and phenolate groups and behave functionally as a dibasic (occasionally tri-basic) acid (Stevenson 1994). As a result, the amount of dissolved organic matter (DOC) in soil pore water tends to increase as the pH increases (Cheshire et al. 1977; Yin et al. 2002). Indeed, addition of dilute NaOH is used as a standard method for extracting humic acids from soil (Parsons 1988; Sparks et al. 1998). Thus, the addition of red mud to soil, which increases the pH, tends to increase the amount of DOC in soil pore waters (Lombi et al. 2002; Lehoux et al. 2013; Rekasi et al. 2013). However, the degree to which a particular soil is affected will depend on the nature of that soil. For example, sandy soils will undergo a greater pH increase, as they lack the intrinsic buffering capacity of a clay soil, and an organic-rich soil will release more DOC into soil pore waters upon soil alkalisation (Lehoux et al. 2013).

In the environment, copper and nickel most commonly occur as M^2+^ cations and are generally more mobile under acidic conditions as they tend to adsorb strongly to minerals at neutral and alkaline pH. Nickel also can form insoluble hydroxides (as Ni(OH)_2_) as pH increases above pH 9 (Richter and Theis 1980; Bradbury and Baeyens 2009). However, Ni(II) and particularly Cu(II) can form strong complexes with organic molecules (Ashworth and Alloway 2004; Baken et al. 2011), especially those that contain carboxyl moieties (Moon and Peacock 2013). High DOC in soil pore waters can therefore have a major impact on the aqueous concentrations of trace metals in circumneutral and alkaline pore waters (Davis 1984; Ashworth and Alloway 2004) despite the tendency of the metal cations to adsorb to minerals at neutral to high pH (Wu et al. 2001, 2002).

Cu is redox sensitive and can be reduced to Cu(I) or Cu(0) in reducing environments forming insoluble oxides, sulphides and elemental Cu (Fulda et al. 2013a; Weber et al. 2009) that can significantly reduce the overall environmental mobility of Cu. The mobility of Ni is less affected by the changes in redox potential found in natural environments, but sorption of Ni to Fe and especially Mn oxides is very important in controlling aqueous Ni concentrations (Peacock and Sherman 2007). Therefore, in reducing environments (where Mn and Fe oxides tend to be dissolved during bioreduction), Ni mobility can be indirectly affected by changes in the availability of sorption sites.

In previous studies of red mud-soil mixtures, increased Cu mobility has been observed in both field trials (Lombi et al. 2002) and laboratory tests (Rekasi et al. 2013). Since the Ajka spill, increased concentrations of both Cu and Ni have been found in the Torna and Marcel Rivers (Nagy et al. 2013). However, if reducing conditions developed with time in water-logged soil, Cu may speciate as less soluble Cu(I) and Cu(0) phases at lower redox potentials, or the reductive dissolution of Fe and Mn oxides may reduce the availability of sorption sites and promote both Cu and Ni solubility. Thus, it is important to understand the potential effects of red mud addition to soils on Cu and Ni mobility under both aerobic and anaerobic conditions.

This paper reports results from long-term aerobic and anaerobic batch experiments that investigate the mobility of Cu and Ni in red mud-contaminated soil-water systems that are representative of soil conditions after the Ajka disaster remediation efforts. The specific objectives of this study were (1) to determine the potential for Cu and Ni release in red mud affected soil-water systems, (2) to determine the effect of complexation with soil derived DOC on metal behaviour as a function of soil type, and (3) to discuss the long-term implications of ploughing in red mud to soils as an emergency remediation method and the potential hazards associated with unremediated wetland areas.

## Materials and methods

### Field sampling and sample handling

**Fig. 1:**
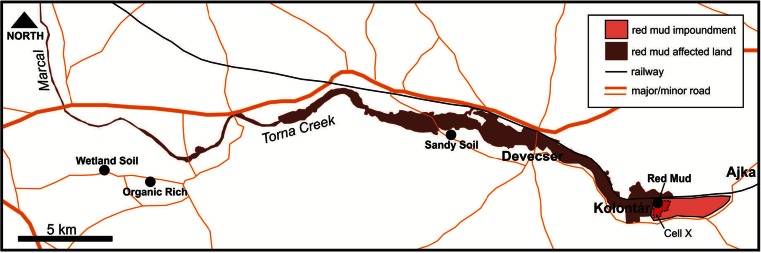
Map of the area affected by the red mud spill in October 2010, showing sampling locations indicated by *filled circles*, from May 2011 taken from Lockwood et al. (2014)

Sampling was undertaken in May 2011 (see Fig. 1). Red mud (RM) was sampled from within Cell X of the Ajka impoundment (Location 47° 05′ 18.48″ N, 17° 29′ 46.77″ E), and red mud leachate was collected from an open leachate pond at the same location. Three uncontaminated soils were sampled from locations in the Torna and upper Marcal river catchments unaffected by the release of red mud in 2010 (see Fig. 1 for sampling locations). Two were agricultural top soils (one was organic-rich (OR), the other a sandy soil (SS)) and the third was collected from 50 cm from below the surface (i.e. beneath the rootlet layer) of a wetland (WL). All samples were stored at 4 °C in polythene containers. The wetland soil was stored anaerobically using Anaerogen™ sachets.

### Long-term batch experiments

Batch microcosm experiments were established under both aerobic and anaerobic conditions. Two different red mud-soil mixtures were prepared for each soil type, and triplicate microcosms were prepared for each condition. A 9 % red mud addition (by dry weight) was chosen as an analogue for where red mud had been ploughed into fields (based on ~5 cm ploughed to a typical depth of 40–50 cm, an approximate 1:10 mixing ratio). The 33 % addition was used as a worst case scenario for any unremediated wetlands. Deionised water was added in a 5:1 ratio to the amount of soil.

Anaerobic experiments were carried out in 120-mL glass serum bottles, which were purged with nitrogen before capping and crimp sealing. Aerobic experiments were carried out in 50-mL polypropylene centrifuge tubes and opened every week day for an hour in order to exchange the experimental headspace with air and prevent anaerobic conditions forming due to microbial respiration. Both aerobic and anaerobic experiments were incubated in the dark at 21 °C and sampled periodically over 100–120 days. During sampling, bottles/tubes were shaken, and 2- to 4-mL aliquots of soil/RM slurry were extracted. Aseptic technique was used where appropriate. Extractions were centrifuged (3 min, 6000 *g*), and the water pH and oxidation/reduction potential (ORP) were determined. The aqueous phase was filtered (0.2 μm) and acidified with 2 % HNO_3_ for ICP-MS analysis. At the end point of each experiment between 6 and 15 mL of slurry that was extracted and centrifuged, the solutions were used for dissolved organic carbon (DOC) analysis and solid phase extraction (SPE) experiments. For each soil type, control experiments without red mud were incubated under anaerobic and aerobic conditions as described above.

### Geochemical methods

ORP (as an indicator for Eh) and pH were measured using a Thermo Scientific Orion Dualstar pH/ISE benchtop meter (pH was calibrated daily at pH values of 4, 7 and 10; a new factory calibrated ORP electrode was used). Aqueous Cu and Ni concentrations were determined using a PerkinElmer Elan DRCII ICP-MS. DOC in end point solutions was determined by a multi N/C® 2100 analyser using thermocatalytic oxidation, MC-NDIR detection analysis. Sequential extractions were performed on triplicate Ajka red mud samples (collected from location K1 in Mayes et al. (2011) in December 2010) following an optimised Tessier procedure (Rauret et al. 1989) that partitioned Cu and Ni into five operationally defined fractions. Extractant pH was checked after each extraction stage and to ensure it conformed to protocol, and Cu and Ni concentrations were determined on an Optima 5300 DV ICP-OES.

### SPE

The end point solutions were passed through Isolute™ C18 non-polar SPE filters (1 g/6 mL) to retain organic substances (and thereby any organically bound metals). These filters were conditioned according to the manufacturer’s instructions, and the solutions acidified to pH 5.5 prior to filtration using HNO_3_ (the optimum pH for maximum organic matter retention by Isolute C18 filters (Thomas 2000)). The filtrates were further diluted with 2 % HNO_3_ for ICP-MS analysis to determine the inorganic/free aqueous metal concentrations. The concentration of organically bound metals was calculated from the difference in aqueous metals concentrations measured before (total [M^+^]) and after SPE (inorganic/free [M^+^]). It should be noted that this is an operationally defined extraction, and a small percentage of metals bound to organic compounds may not be retained by C18 filters and thus appear in the aqueous fraction. The percentages calculated from this method will therefore be used as an indicator for the amount of organically bound metals.

## Results

### Sample characterisation

**Fig. 2:**
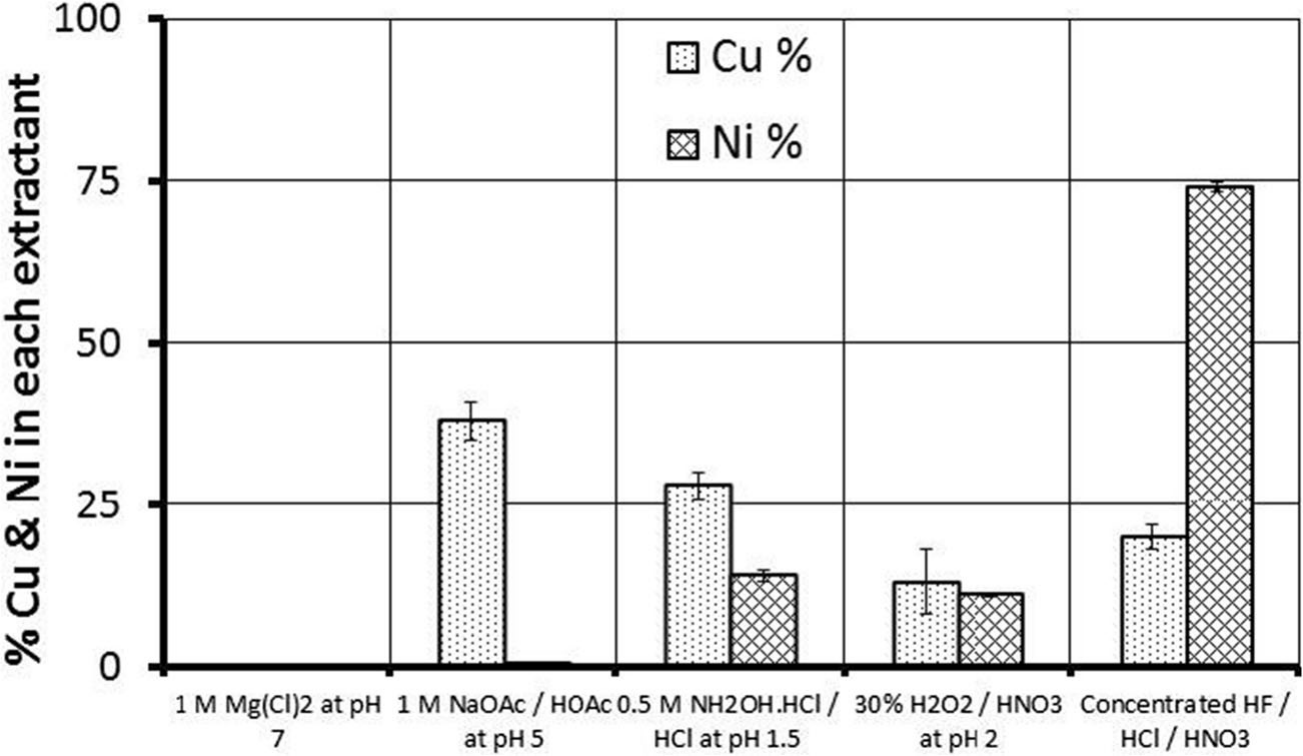
Sequential extraction data from triplicate red mud samples (from location K1, Mayes et al. (2011))

The RM and the three different soil samples have been fully described previously (Lehoux et al. 2013) and are summarised in Table 1. X-ray fluorescence (XRF) analysis of the samples is presented in Table A (Online Resource). Briefly, the RM mineral content was dominated by hematite (Fe_2_O_3_), calcite (CaCO_3_), magnetite (Fe_3_O_4_), cancrinite (Na_6_CaAl_6_Si_6_(CO_3_)O_24_ · 2H_2_O) and hydrogarnet (Ca_3_AlFe(SiO_4_)(OH_8_) with residual boehmite (γ-AlOOH) and gibbsite (Al(OH_3_)) phases, which is very similar to other red mud from the breach area (Gelencser et al. 2011; Burke et al. 2012). The concentrations of Cu and Ni in red mud were 104 and 361 mg kg^−1^, respectively. In sequential extractions on the red mud (see Fig. 2 and Online Resource Table B), an extremely low proportion of the Cu and Ni was exchangeable with Mg^2+^. Most (~80 %) of the Cu present was progressively leached from the red mud by a series of weakly acidic (pH 1.5–5) leaching solutions. The majority (~75 %) of the Ni, however, was only released from the red mud by total digestion.

**Table 1:** Red mud and soil characterisation

Soil sample	Soil type	Total Cu (mg kg^−1^)^a^	Total Ni (mg kg^−1^)^a^	pH	% 0.5 M HCl extractable iron as Fe(II)	Major minerals present	TOC (%)	BET surface area (m^2^ g^−1^)
Red mud	n/a	104	361	12.3	22 (±3)	Hematite, calcite, hydrogarnet, boehmite, cancrinite	0.23	14.40 (±0.07)
Soil OR	Sandy clay loam	12	23	7.0	9.6 (±2)	Quartz, albite, clinochlore, muscovite, ilite	4.15	1.78 (±0.20)
Soil WL	Sandy clay loam	6	14	7.9	87.3 (±14)	Quartz, albite, clinochlore, muscovite, ilite	1.14	2.61 (±0.01)
Soil SS	Sandy loam	2	5	7.9	4.6 (±2)	Quartz, albite, clinochlore, ilite	0.74	0.94 (±0.01)

All data except for total Cu and total Ni was taken from Lehoux et al. (2013). Note that the samples’ names used here differ to those in Lehoux et al. (2013), for clarification soil SS was previously named H1, soil OR was named H2 and soil WL was named H3

All three soils had similar mineralogy, with quartz as the dominant mineral, and feldspars and clays also present. The principal differences between them were in the organic carbon contents, and the proportions of the 0.5 N HCl extractable iron in the Fe(II) oxidation state (Table 1). Soil OR had the highest concentrations of Cu (12 mg kg^−1^) and Ni (23 mg kg^−1^). Soil WL had 6 Cu and 14 mg kg^−1^ Ni. Soil SS had the lowest concentrations of Cu (2 mg kg^−1^) and Ni (5 mg kg^−1^). PCA analysis (Fig. A, Online Resource) shows that these soils were similar to other reference soils from the area that were unaffected by the 2010 RM spill (Mayes et al. 2011; Lehoux et al. 2013).

### Effect of RM addition on microcosm pH and DOC

**Fig. 3:**
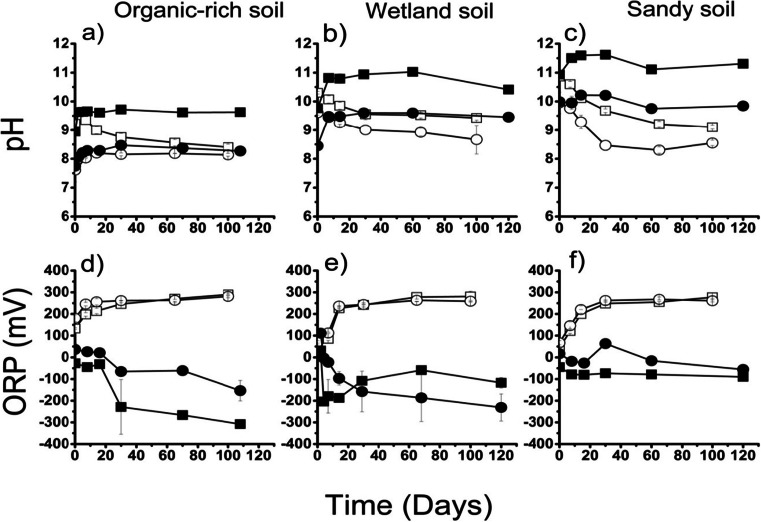
pH and ORP in anaerobic and aerobic RM-amended experiments over time. *Black* = anaerobic, *white* = aerobic. *Square* = 33 % RM-amended, *circle* = 9 % RM-amended. *Error bars* are 1σ of triplicate results (where not shown, errors are within the symbol size). Graphs for equivalent unamended controls can be viewed in the Online Resource

The pH of the soil-only control experiments was very similar under both anaerobic and aerobic conditions and was between pH 7 and 8 for each soil type (Fig. B, Online Resource). The pH of the anaerobic and aerobic experiments developed differently over time. The pH of the RM-amended anaerobic systems (Fig. 3a–c) remained relatively constant after an initial equilibration period. For each soil type, the 33 % RM-amended systems had the higher pH, with final pH values of about 9.5, 10.5 and 11.5 for the OR, WL and SS soils, respectively. The 9 % RM-amended experiments also became more alkaline than the soil-only controls (final pH values ~8.5, 9.5 and 10 for the OR, WL and SS soils, respectively). The initial pH values of the aerobic experiments were very similar to those of the equivalent anaerobic experiments; however, the pH of the aerobic experiments containing red mud gradually decreased over time. The final pH values of the aerobic 9 % RM tests were pH ~8 with OR soil and just above pH 8.5 with WL and SS soil. The final pH values of the aerobic 33 % RM tests were between a half and one pH unit higher than the equivalent 9 % RM tests. In all anaerobic experiments, the ORP decreased to between −100 and −300 mV. In the aerobic experiments, it increased to ~ +250 mV (Fig. 3d–f).

For each soil, the amount of DOC in solution at the end of testing increases with the RM loading in both anaerobic and aerobic conditions, with significantly higher DOC concentrations in the anaerobic systems than in the equivalent aerobic systems (this is most apparent in the OR Soil, 33 % RM experiment, where the DOC concentration is ~850 mg L^−1^ in the anaerobic system, but only ~82 mg L^−1^ in the aerobic system). Comparison of the three soils indicates that the DOC concentrations in OR soil tests were higher than in the equivalent WL or SS soil tests (the WL and SS soils exhibited broadly similar DOC concentrations in equivalent tests) (Table 2).

**Table 2:** DOC concentrations (mg L^−1^) at experimental end points

	Soil OR DOC (mg L^−1^)		Soil WL DOC (mg L^−1^)		Soil SS DOC (mg L^−1^)	
	Anaerobic	Aerobic	Anaerobic	Aerobic	Anaerobic	Aerobic
Unamended Control	81 (±8)	11 (±2)	21 (±2)	8 (±5)	NR	13 (±3)
9 % RM Addition	150 (±3)	31 (±4)	95 (±16)	37 (±1)	149 (±22)	22 (±4)
33 % RM Addition	850 (±6)	82 (±8)	209 (±39)	67 (±3)	262 (±4)	58 (±7)

*NR* not analysed

### Mobilisation of Cu and Ni from RM-affected soil-water systems

**Fig. 4:**
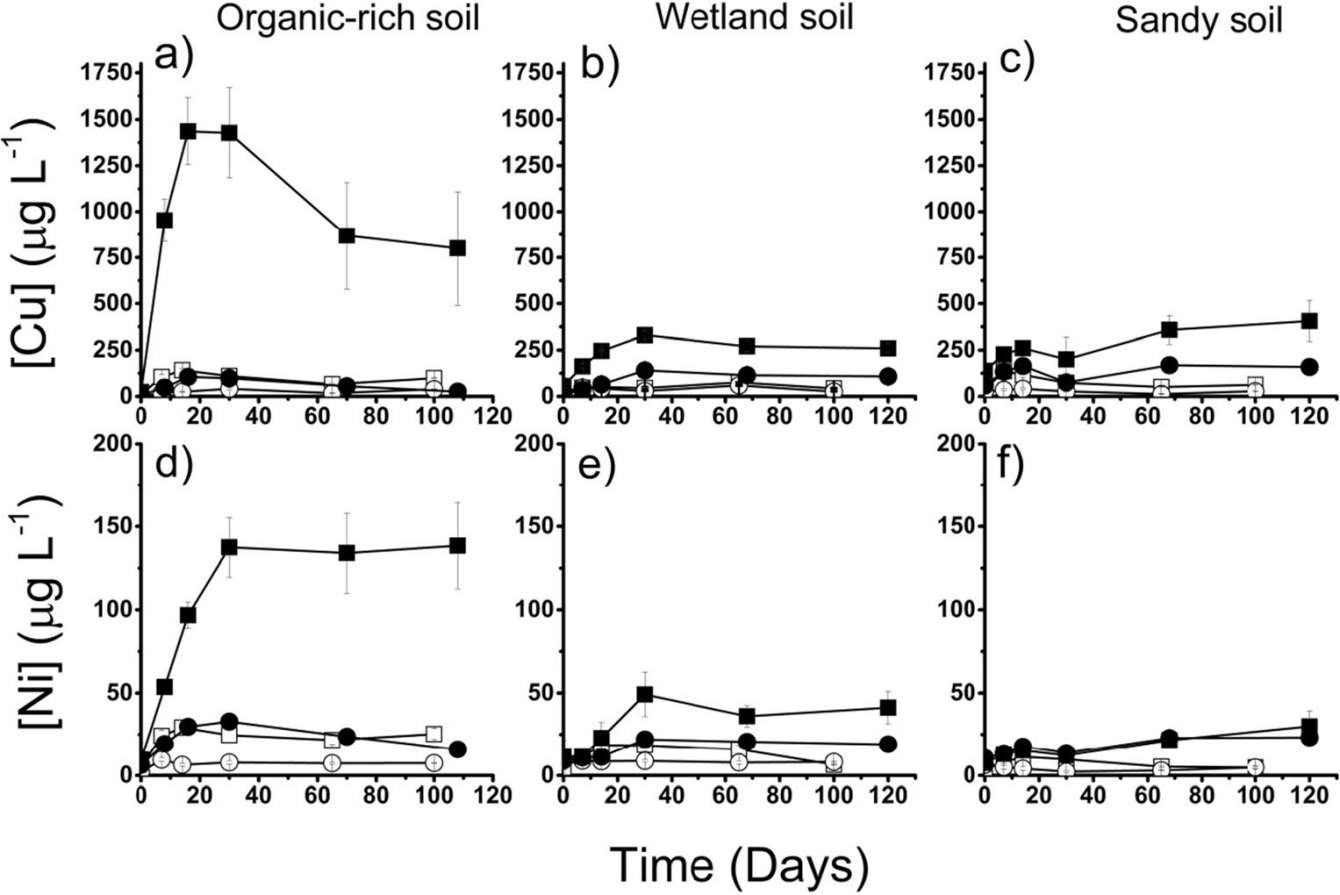
Evolution of aqueous Cu and Ni over time in anaerobic and aerobic RM-amended experiments. *Black* = anaerobic, *white* = aerobic. *Square* = 33 % RM-amended, *circle* = 9 % RM-amended. *Error bars* are 1σ of triplicate results (not shown, errors are within the symbol size). Graphs for equivalent unamended controls can be viewed in the Online Resource (Fig. C)

The aqueous Cu concentrations in the anaerobic soil-only controls were all ~15 μg L^−1^ and did not vary much with time (see Online Resource, Fig. C). After an equilibration period, the aqueous Cu concentrations in the anaerobic RM-amended experiments were significantly higher than in the controls (Fig. 4a–c) and increased with the amount of RM added (a slight exception is the OR soil amended with 9 % RM, where the final concentration was only slightly higher than the control, <25 μg L^−1^). The highest aqueous Cu concentration of ~1450 μg L^−1^ was recorded after 30 days with the OR soil amended with 33 % RM, although this concentration subsequently decreased with time to ~850 μg L^−1^. The aqueous Cu concentrations in the aerobic controls were generally higher than in the anaerobic controls (typically about 20 μg L^−1^) and showed more variation but no trend with time. The aqueous Cu concentrations in the aerobic RM-amended experiments were generally slightly higher than in the soil-only controls (25–100 μg L^−1^) but significantly lower than in the equivalent anaerobic experiments.

The aqueous Ni concentrations in the anaerobic soil-only controls were all <10 μg L^−1^ and did not vary much with time. In the anaerobic RM-amended experiments, the aqueous Ni concentration was higher than in the anaerobic controls and followed a similar pattern to the evolution of aqueous Cu (although concentrations were about five times smaller). In the anaerobic systems amended with 33 % RM, the final Ni concentrations were 150, 42 and 34 μg L^−1^ for the OR, WL and SS soils, respectively. The aqueous Ni concentrations in the aerobic controls were generally slightly lower than in the anaerobic controls (typically <7 μg L^−1^). In the aerobic systems amended with 33 % RM, the final Ni concentrations were 25, 6 and 5 μg L^−1^ for the OR, WL and SS soils, respectively (Fig. 4d–f). Thus, aqueous Ni concentrations in the aerobic RM-amended experiments were similar to those of the equivalent controls, with the exception of the OR Soil amended with 33 % RM, which was four times higher.

### Organically bound Cu and Ni

In all the systems except the WL aerobic control experiment, a significant proportion of the aqueous Cu was organically complexed (Table 3(A), (B)). This proportion was generally higher in the experiments where red mud was added. In the WL and SS systems, there was generally slightly more organically complexed Cu in the anaerobic system than in the aerobic systems but very little difference in the OR systems. No organically complexed Ni was found in either set of controls experiments. However, organically complexed Ni was found in aerobic experiments with OR and WL soil where 33 % RM was added, and significant amounts of organically complexed Ni were found in all the anaerobic experiments where RM was added.

**Table 3:** % Organically bound aqueous metals from anaerobic (A) and aerobic (B) end point solutions

A	% Cu			% Ni		
	Soil OR	Soil WL	Soil SS	Soil OR	Soil WL	Soil SS
Unamended controls	37 (±13)	64 (±2)	NR	0^a^	0^a^	NR
9 % RM addition	73 (±6)	91 (±1)	57 (±9)	28 (±6)	34 (±9)	14 (±2)
33 % RM addition	70 (±7)	80 (±3)	73 (±7)	46 (±10)	39 (±7)	48 (±13)
B	% Cu			% Ni		
	Soil OR	Soil WL	Soil SS	Soil OR	Soil WL	Soil SS
Unamended controls	65 (±3)	4 (±4)	47 (±8)	0^a^	0^a^	0^a^
9 % RM addition	72 (±2)	55 (±4)	60 (±3)	0^a^	0^a^	0^a^
33 % RM addition	75 (±2)	32 (±1)	55 (±2)	55 (±5)	12 (±10)	0^a^

*NR* not analysed

^a^Inorganic/free[M^+^] was ≥ total [M^+^]; therefore, organically bound [M^+^] was assumed to be zero

## Discussion

### Effect of red mud addition on soil pH and DOC

When red mud was added to the anaerobic soil-water systems, there was an immediate increase in pH which was related to the red mud loading, after which the pH remained relatively constant throughout the incubation period. The change in pH is due to NaOH in the red mud, but it varied with soil type due to differences in buffering capacity. The pH buffering capacities of these soils result from the clay minerals and particularly the organic matter they contain (Lehoux et al. 2013). Alkaline fluctuations are usually buffered by deprotonation reactions (Celik 2004; Stevenson 1994):

1 $$ \left[\mathrm{Clay}-\mathrm{M}-\mathrm{O}\mathrm{H}\right]+\left[\mathrm{O}{\mathrm{H}}^{-}\right]\ \to \left[\mathrm{Clay}-\mathrm{M}-{\mathrm{O}}^{-}\right]+{\mathrm{H}}_2\mathrm{O} $$

2 $$ \left[R-\mathrm{CO}\mathrm{O}\mathrm{H}\right]+\left[\mathrm{O}{\mathrm{H}}^{-}\right]\to \left[\mathrm{R}-\mathrm{C}\mathrm{O}{\mathrm{O}}^{-}\right]+{\mathrm{H}}_2\mathrm{O} $$

The dissolution of amorphous and poorly crystalline silica starts to become important in systems with a pH above 9.8 (Langmuir 1997), so this may also affect the buffering capacity of systems with a high pH and a high Si content (reaction 3 (Langmuir 1997)).

3 $$ \left[\mathrm{S}\mathrm{i}{\mathrm{O}}_2\right]+2\left[{\mathrm{H}}_2\mathrm{O}\right]\to \left[{\mathrm{H}}_2\mathrm{S}\mathrm{i}{{\mathrm{O}}_4}^{-}\left]+\right[{\mathrm{H}}^{+}\right] $$

Thus, at each red mud loading, the pH value of the SS system > the WL system > the OR system.

In aerobic systems, the initial increase in pH upon red mud addition was similar to that observed in the anaerobic systems; however, there was a subsequent gradual decrease of pH. The latter trend can be explained by carbonation by atmospheric CO_2_, as formation of aqueous carbonate species consumes OH^−^ resulting in a reduction in pH (Schwab et al. 2006). Carbonate ions tend to react with any divalent cations present (e.g. Ca(II) and Mg(II) from either the soils or the red mud) to precipitate carbonate minerals. Thus, the final pH of all the systems buffers to between 8.0 and 9.5 regardless of soil type. The lower final pH value of the aerobic experiments resulted in lower measured DOC concentrations in all systems compared to the equivalent anaerobic experiments.

For the anaerobic experiments, the DOC released from the OR systems was between three and four times higher than that released from the WL or SS systems at the same level of red mud loading. This suggests that the amount of soil organic matter present is the main factor controlling DOC release in these experiments (the OR soil has 4.15 % TOC compared to 1.14 and 0.74 % in the WL and SS soils, respectively). The higher initial pH values of the wetland and sandy soils compared with the OR soil (7.9, 7.9 and 7.0 respectively) may also have meant that SOM in these soils contained a lower proportion of humic acids. Humic acids are the main alkaline soluble component of SOM. As conditions become increasingly basic, the humic acids dissociate, and therefore, their solubility is increased (Stevenson 1994). Under aerobic conditions, the DOC concentration in the batch experiment end point solutions was much lower than in the equivalent anaerobic tests (up to 10 times less at the same red mud loading). This was most likely to be due to the lower pH of the aerobic systems.

### Controls on copper release

Cu solubility in soils is often controlled by pH. At neutral and high pH values, Cu^2+^ adsorbs strongly to negatively charged mineral surfaces, and solution concentrations are low (Peacock and Sherman 2004). In the presence of DOC, Cu can form stable aqueous organo-metallic complexes thus increasing Cu concentrations in neutral and alkaline conditions (Davis 1984; Wu et al. 2002). It is therefore important to establish the main control of Cu mobilisation in these experiments where both pH and DOC are key variables.

In experiments with a red mud addition, there was no statistically significant correlation (Pearson’s *r* = <0.5, *p* = > 0.1; Table 4) between aqueous Cu concentrations and pH in either the anaerobic or aerobic systems. However, there was a significant correlation (Spearman’s *r* = > 0.8, *p* = <0.001) between aqueous Cu concentrations and DOC concentrations in both systems. Also, as a significant proportion of dissolved Cu was found to be organically complexed (the fraction retained by the SPE filters), this suggests that dissolution of SOM (specifically the humic acid fraction) is principal control on Cu solubility in both anaerobic and aerobic experiments. These findings are in agreement with previous work, which found that ~62 % of Cu mobilised from soils amended with red mud was associated primarily with OM (Lombi et al. 2002).

**Table 4:** Spearman’s rank correlation values for aqueous Cu and Ni concentrations vs pH or DOC for red mud-amended experiments after 100–120 days incubation

Determinant	Experimental conditions			
	Anaerobic		Aerobic	
	*r*_s_ value	*p* value	*r*_s_ value	*p* value
[Cu] vs				
DOC	0.86	<0.001	0.79	<0.001
pH	0.50	0.038	0.16	0.501
[Ni] vs				
DOC	0.78	<0.001	0.45	0.06
pH	0.43	0.08	−0.46	0.05

Degrees of freedom was 16 for all correlations (Wessa 2014). Regarding the interpretation of Spearman’s *r*_s_ and *p* values, −*r*_s_ can be a value between +1 to −1. An *r*_s_ value of 1.0 indicates a perfect linear association, either positive or inverse. *r*_s_ of 0 indicates that there is no association, and the closer to 0 the value then the weaker the association. This must be interpreted together with the *p* value. If *p* = <0.05, the relationship is statistically significant; if *p* = > 0.05, there is no statistical significance)

For all the tests on the three soils, the amount of Cu released to solution is less than the amount of Cu that was present in the soil prior to the addition of red mud (the highest proportion was mobilised in the anaerobic OR 33 % RM test, where it is equivalent to 90 % of the Cu originally associated with the soil). The sequential extraction data (see Fig. 2 and Table B, Online resource) indicates that very little Cu is adsorbed to the red mud in exchangeable surface sites (~0.1 mg kg^−1^). The fact that most of the Cu is released to solution by weak acid may be indicative of Cu present as inner-sphere surface complexes on red mud minerals (i.e. Cu(II) starts to be released from surfaces when the pH is below ~6, for example, the sorption edge value for Cu on ferrihydrite and hematite is ~ pH 5.2 (Langmuir 1997; Christl and Kretzschmar 2001)) or that Cu is incorporated in solid phases that are dissolved by the leaching solutions. In the presence of soluble organic compounds, Cu(II) affinity for surfaces is reduced for all pH values (Ali and Dzombak 1996); therefore, it is possible that the high DOC present in red mud-affected soils may promote release of surface bound Cu(II) present in red mud. However, it is also possible to account for all of the Cu released to solution in these experiments by considering just the Cu originally present in the soils (i.e. the maximum Cu concentration observed in experiments is equivalent to ~30–100 % of the original soil associated Cu). In this case, SOM-bound Cu would remain complexed to organic compounds solubilised by the high pH.

**Fig. 5:**
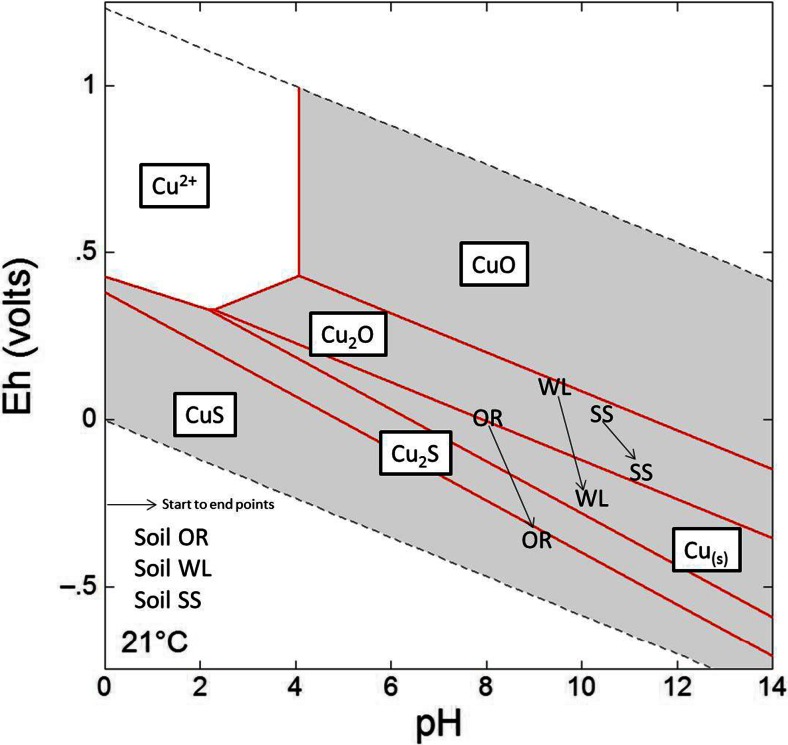
Eh/pH conditions calculated for 33 % RM-amended anaerobic experiments superimposed on an Eh/pH Cu species predominance and relative mineral stability diagram calculated using Geochemists Workbench® for *t* = 21 °C/*P* = 1 at, for the system Cu-O-H-SO_4_^2−^ with log ∑Cu/m = −.3256, log ∑SO_4_^2−^/m − .3646 and a[H_2_O_(aq)_] = 1. *Points plotted* show geochemical conditions at day 0 and experimental end points

The aqueous Cu concentration in the OR 33 % RM experiment peaked after 15 days and then decreased with time (Fig. 4a). As the pH did not vary significantly after day 15, this decrease in aqueous Cu concentration is unlikely to be associated with humate solubility. However, the ORP decreased rapidly between 15 and 30 days (Fig. 3d), and sulphate was removed from aqueous solution (see Online Resource, Fig. D) suggesting the occurrence of sulphate reduction. Cu(II) species are the most stable Cu species under oxidising conditions, but Cu(I) and Cu(0) species are formed under more reducing conditions (Leckie and Davis 1979). Both Cu(II) and Cu(I) can form stable complexes with organic matter, but inorganic sulphide is thought to outcompete OM for Cu(I) in sulphide-rich environments (Fulda et al. 2013b). The Eh/pH diagram for relevant inorganic Cu species (Fig. 5) confirms that the anaerobic OR 33 % RM test reached an Eh/pH state where sulphide can compete for the Cu. There is also evidence for Cu removal from solution associated with the onset of sulphate reduction (see Online Resource, Fig. D) in the data from the OR 9 % RM experiment (Figs. 4a and 3d: note that the ORP for sulphate reduction is higher at pH 8 than at pH 9.5). By the end of this test, there was very little Cu in solution despite an elevated DOC in comparison with the soil-only control (i.e. the end-point of this test did not follow the trend of increasing aqueous Cu concentration with increasing DOC concentration). Thus, where the soil pH and the availability of organic matter favour sulphate reduction, the formation of sulphides can curtail Cu mobilisation by aqueous humates and therefore lower aqueous Cu concentrations.

### Controls on nickel release

Nickel solubility decreases with increasing pH in inorganic systems due to the increased sorption of Ni(II) species on negatively charged mineral surfaces (Richter and Theis 1980), and the tendency of Ni(II) species [Ni(OH)^+^ and Ni(OH)_2_] to form hydrolysed surface complexes or precipitates when pH >9 (for concentrations <1 × 10^−3^ M) (Bradbury and Baeyens 2009; Peacock and Sherman 2007). Whilst Ni speciation is not affected by redox chemistry (Brookins 1988), it is known to form stable complexes with OM which can increase the solubility of Ni in some systems (Richter and Theis 1980; Achterberg et al. 1997; Ashworth and Alloway 2004). As with Cu, correlations revealed (Table 4) that in experiments with a red mud addition, there was some correlation (Spearmans’s *r*_s_ = > 0.4, *p* = <0.06) between aqueous Ni concentrations and DOC concentrations in both the anaerobic or aerobic systems (and no statistically significant correlation with pH). However, the significance level (*p* = <0.06 vs. <0.001) and the strength (*r*_s_ = > 0.4 vs. >0.8) of the correlation was not as high for Ni as was previously seen for Cu. This maybe because a smaller fraction of Ni was found to be organically complexed (i.e. retained by the SPE filters) in anaerobic experiments than for Cu. In aerobic experiments, organically bound Ni was only detected in the OR and WL experiments where there was a 33 % addition of red mud.

It is also possible to account for all of the Ni released to solution by considering only the Ni originally present in the soils (i.e. the maximum Ni concentration observed in experiments is only 2–3 % of the original soil-associated Ni). In contrast to Cu, most of the Ni present in red mud was associated with residual phases in the sequential extraction (i.e. those dissolved by HF/aqua regia). For red mud, this includes residual iron oxides including hematite (Mayes et al. 2011). Ni readily substitutes into hematite (Singh et al. 2000); therefore, it is very unlikely that the Ni present in red mud can be mobilised due to the presence of DOC in solution and SOM-associated Ni most probably dominates the Ni released in red mud affected soils.

**Fig. 6:**
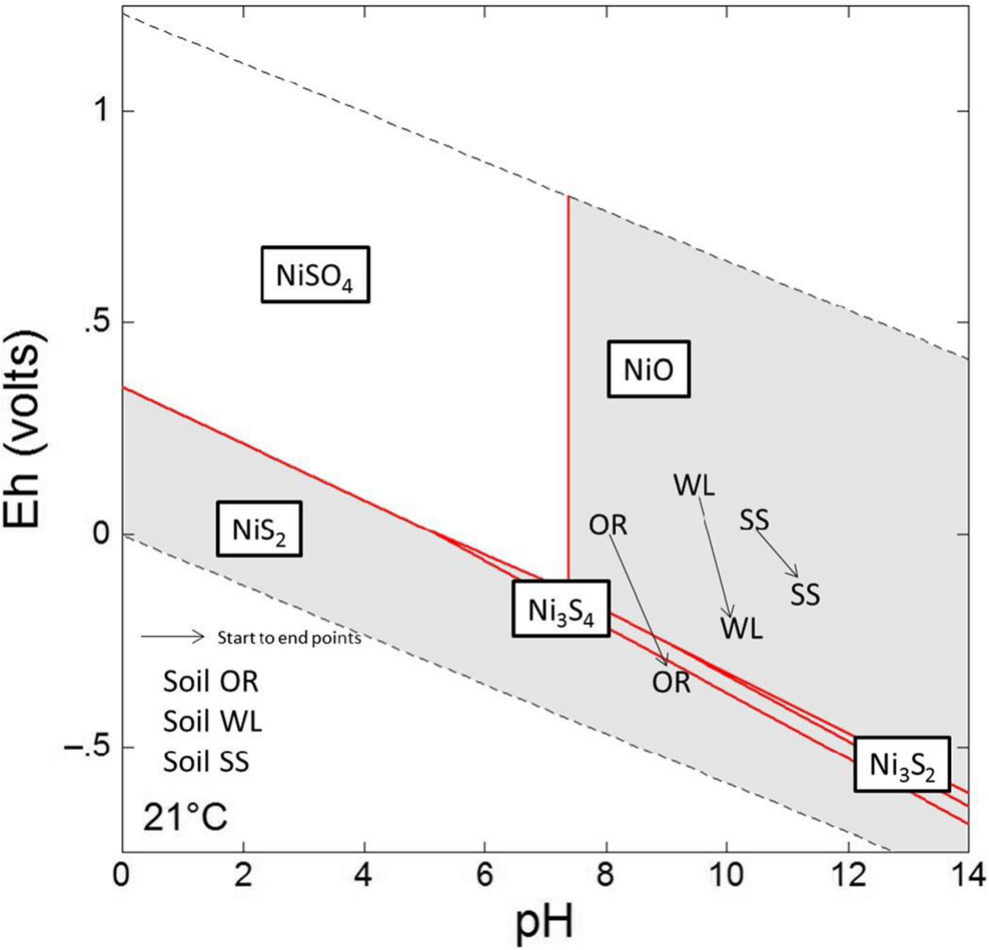
Eh/pH conditions calculated for 33 % RM-amended anaerobic experiments superimposed on an Eh/pH Ni species predominance and relative mineral stability diagram calculated using Geochemists Workbench® for *t* = 21 °C/*P* = 1 at, for the system Ni-O-SO_4_^2−^ with log ∑Ni/m = −.3256, log ∑SO_4_^2−^ −.3646 and a[H_2_O_(aq)_] = 1. *Points plotted* show geochemical conditions at day 0 and experimental end points

An Eh/pH diagram for relevant inorganic Ni species is shown in Fig. 6, with the Eh/pH states of the anaerobic tests annotated on it. This figure shows that the thermodynamically stable Ni-containing phase is NiO without SOM present, except where strongly reducing conditions developed towards the end of the anaerobic OR tests, where sulphidic phase [NiS_2_] should be the stable. However, unlike Cu, there is little change in the aqueous Ni concentration. This may be because a lower proportion of the Ni is organically bound or may reflect the significantly lower aqueous nickel concentrations (Cu concentrations are 10 times higher in this experiment, Fig. 4). These data indicate that DOC is an important mechanism for controlling Ni mobility, especially when DOC concentrations are elevated; although compared to Cu, the affinity for Ni to OM is weaker (Ashworth and Alloway 2004). Furthermore, most of the Ni present in red mud is not likely to easily mobilised, which has resulted in lower aqueous Ni concentrations despite all three systems containing higher total nickel concentrations when compared to copper.

### Implications for red mud affected soils and wetlands

The ploughing of red mud into soil is an effective short-term measure for the prevention of dust formation following a spill to land (Gelencser et al. 2011). This addition of red mud to soil increases soil pH which in turn releases alkaline-soluble components of SOM into the soil pore water along with any metals complexed to the mobilised SOM. If metals associated with the red mud also become complexed to SOM, this could cause further mobilisation of contaminant metals. In soils taken from the Torna and upper Marcal River, catchments near to, but unaffected by, the Ajka red mud spill Cu were more readily mobilised by contact with red mud than Ni. This was despite both the soils and the red mud containing more Ni than Cu. Also, a higher proportion of Cu in the aqueous phase was organically complexed than Ni in all three soils under all the conditions investigated. Thus, the difference in Cu and Ni mobilisation by red mud addition is most probably associated with the higher affinity of Cu to form OM complexes and by the fact that the red mud associated Cu was much more likely to be mobilised than Ni.

If soils that received red mud are in contact with atmosphere (i.e. are aerobic; as would be expected for most agricultural soils, except during seasonal flooding), the pH of alkaline pore water is reduced by carbonation reactions; therefore, organic matter dissolution is reduced and the associated Cu/Ni mobility is lowered. Therefore, if agricultural soils are expected to remain aerobic over the long-term, then Cu and Ni mobilisation will be limited especially for smaller red mud additions.

However, where soils are not in contact with atmosphere (i.e. are anaerobic; as would be expected for a permanemtly flooded wetland environment), soil pH will remain high pH, and elevated Cu and Ni concentrations in the pore water may persist in the long-term. The potential risks are therefore higher in low lying riparian wetlands where large amounts of red mud may have been deposited. Cu and Ni mobility will be higher in anaerobic than aerobic soils, but if soil conditions become sufficiently reducing (that sulphate reduction occurs), Cu is removed from solution by reduction of Cu(II), decomplexation from SOM and the formation of insoluble Cu-sulphide mineral phases. However, in this study, this only occurred in experiments with high organic matter content and where potentially there could be very high Cu concentrations in solution.

## Conclusion

The amendment of a range of soils with red mud resulted in mobilisation of Cu and Ni to solution. The high pH conditions imposed by the red mud on soils resulted in higher concentrations of DOC and organically complexed metals. Therefore, soils with higher organic matter content experienced larger metal releases. In all cases, Cu was preferentially released with respect to Ni, despite the higher overall abundance of Ni in solids, due to a higher affinity of Cu to form organic complexes. Aerobic conditions resulted in lower pH, due to carbonation reactions, lower overall DOC concentrations and lower Cu and Ni release. Carbonation was absent under anaerobic condition, and higher Cu and Ni concentration persisted in these experiments. In experiments where sulphate reduction was observed, Cu(II) reduction resulted in the partial removal of Cu from solution. Overall, the addition of high concentrations red mud to soils should be avoided, especially to organic-rich soils in permanently anaerobic environments.

## Electronic supplementary material

Below is the link to the electronic supplementary material.

ESM 1 (DOCX 707 kb)

## References

[CR1] Achterberg EP (1997). Speciation and cycling of trace metals in Esthwaite Water: a productive English lake with seasonal deep-water anoxia. Geochim Cosmochim Acta.

[CR2] Adam J, Banvolgyi G, Dura G, Grenerczy G et al (2011) The Kolontar report: causes and lessons from the red mud disaster. Sustainable Development Committee of the Hungarian Parliament, Budapest

[CR3] Ali MA, Dzombak DA (1996). Effects of simple organic acids on sorption of Cu^2+^ and Ca^2+^ on goethite. Geochim Cosmochim Acta.

[CR4] Anton A (2012). Modelling the Potential Effects of the Hungarian Red Mud Disaster on Soil Properties. Water Air Soil Pollut.

[CR5] Ashworth DJ, Alloway BJ (2004). Soil mobility of sewage sludge-derived dissolved organic matter, copper, nickel and zinc. Environ Pollut.

[CR6] Baken S (2011). Metal Complexation Properties of Freshwater Dissolved Organic Matter Are Explained by Its Aromaticity and by Anthropogenic Ligands. Environ Sci Technol.

[CR7] Bradbury MH, Baeyens B (2009). Sorption modelling on illite Part I: Titration measurements and the sorption of Ni, Co, Eu and Sn. Geochim Cosmochim Acta.

[CR8] Brookins D (1988). Eh, pH diagrams for geochemistry.

[CR9] Burke IT (2012). Speciation of arsenic, chromium, and vanadium in red mud samples from the ajka spill site, hungary. Environ Sci Technol.

[CR10] Burke IT (2013). Behavior of Aluminum, Arsenic, and Vanadium during the Neutralization of Red Mud Leachate by HCl, Gypsum, or Seawater. Environ Sci Technol.

[CR11] Celik MS, Wypyck F, Satyanarayana KG (2004). Electrokinetic behaviour of clay surfaces. Clay surfaces: fundamentals and applications.

[CR12] Cheshire MV (1977). METAL DISTRIBUTION AND NATURE OF SOME CU, MN AND V COMPLEXES IN HUMIC AND FULVIC-ACID FRACTIONS OF SOIL ORGANIC-MATTER. Geochim Cosmochim Acta.

[CR13] Christl I, Kretzschmar R (2001). Interactions of Cu and fulvic acid at the hematite-water interface. Geochim Cosmochim Acta.

[CR14] Davis JA (1984). Complexation of trace metals by adsorbed natural organic matter. Geochim Cosmochim Acta.

[CR15] Enserink M (2010). ENVIRONMENT After Red Mud Flood, Scientists Try to Halt Wave of Fear and Rumors. Science.

[CR16] Fulda B (2013). Redox transformation, solid phase speciation and solution dynamics of copper during soil reduction and reoxidation as affected by sulfate availability. Geochim Cosmochim Acta.

[CR17] Fulda B (2013). Copper Redox Transformation and Complexation by Reduced and Oxidized Soil Humic Acid. 1. X-ray Absorption Spectroscopy Study. Environ Sci Technol.

[CR18] Gelencser A (2011). The red mud accident in Ajka (Hungary): characterization and potential health effects of fugitive dust. Environ Sci Technol.

[CR19] Grafe M (2011). Bauxite residue issues: III. Alkalinity and associated chemistry. Hydrometallurgy.

[CR20] Hind AR (1999). The surface chemistry of Bayer process solids: a review. Colloids Surf A Physicochem Eng Asp.

[CR21] Klebercz O (2012). Ecotoxicity of fluvial sediments downstream of the Ajka red mud spill, Hungary. J Environ Monitor JEM.

[CR22] Langmuir D (1997). Aqueous environmental geochemistry.

[CR23] Leckie JO, Davis JA, Nriagu JO (1979). Aqueous Environmental Chemistry of Copper. Copper in the Environment Part 1: Ecological Cycling.

[CR24] Lehoux AP (2013). Gypsum addition to soils contaminated by red mud: implications for aluminium, arsenic, molybdenum and vanadium solubility. Environ Geochem Health.

[CR25] Liu Y (2007). Characterization of red mud derived from a combined Bayer Process and bauxite calcination method. J Hazard Mater.

[CR26] Lockwood CL (2014). Mobilisation of arsenic from bauxite residue (red mud) affected soils: effect of pH and redox conditions. Appl Geochem.

[CR27] Lombi E (2002). In situ fixation of metals in soils using bauxite residue: chemical assessment. Environ Pollut.

[CR28] Mayes WM (2011). Dispersal and Attenuation of Trace Contaminants Downstream of the Ajka Bauxite Residue (Red Mud) Depository Failure, Hungary. Environ Sci Technol.

[CR29] Milacic R (2012). Environmental impact of toxic elements in red mud studied by fractionation and speciation procedures. Science of the Total Environment.

[CR30] Moon EM, Peacock CL (2013). Modelling Cu(II) adsorption to ferrihydrite and ferrihydrite-bacteria composites: deviation from additive adsorption in the composite sorption system. Geochim Cosmochim Acta.

[CR31] Nagy AS (2013). Trace metal and metalloid levels in surface water of the Marcal River before and after the Ajka red mud spill, Hungary. Environ Sci Pollut Res.

[CR32] Parsons JW, Frimmel FH, Christman RF (1988). Isolation of humic substances from soils and sediments. Humic substances and their role in the environment.

[CR33] Peacock CL, Sherman DM (2004). Copper(II) sorption onto goethite, hematite and lepidocrocite: a surface complexation model based on ab initio molecular geometries and EXAFS spectroscopy. Geochim Cosmochim Acta.

[CR34] Peacock CL, Sherman DM (2007). Sorption of Ni by birnessite: equilibrium controls on Ni in seawater. Chem Geol.

[CR35] Power G (2011). Bauxite residue issues: I. Current management, disposal and storage practices. Hydrometallurgy.

[CR36] Rauret G (1989). OPTIMIZATION OF TESSIER PROCEDURE FOR METAL SOLID SPECIATION IN RIVER SEDIMENTS. Int J Environ Anal Chem.

[CR37] Reeves HJ, Wealthall G, Younger PL (2011) Advisory visit to the bauxite processings tailings dam near Ajka, Vesprem County, western Hungary. British Geological Survey, Keyworth

[CR38] Rekasi M (2013). Effects of leaching from alkaline red mud on soil biota: modelling the conditions after the Hungarian red mud disaster. Chem Ecol.

[CR39] Renforth P (2012). Contaminant mobility and carbon sequestration downstream of the Ajka (Hungary) red mud spill: the effects of gypsum dosing. Sci Total Environ.

[CR40] Richter RO, Theis TL (1980) Nickel speciation in a soil/water system. In: Nriagu JO (ed) Nickel in the Environment

[CR41] Rubinos DA, Barral MT (2013). Fractionation and mobility of metals in bauxite red mud. Environ Sci Pollut Res Int.

[CR42] Ruyters S (2011). The red mud accident in Ajka (Hungary): plant toxicity and trace metal bioavailability in red mud contaminated soil. Environ Sci Technol.

[CR43] Schwab AP (2006). Characteristics of blast furnace slag leachate produced under reduced and oxidized conditions. J Environ Sci Health A Toxicol Hazard Subst Environ Eng.

[CR44] Secretariat of the Basel Convention (2011) Basel Convention of the control of transboundary movements of hazardous waste and their disposal. Protocol on liability and compensation for damage resulting from transboundary movements of hazardous wastes and their disposal. Texts and Annexes. Annex IX, List B2, B2110, pg 81

[CR45] Singh B (2000). Structural chemistry of Fe, Mn, and Ni in synthetic hematites as determined by extended X-ray absorption fine structure spectroscopy. Clay Clay Miner.

[CR46] Sparks DL, Scheidegger AM, Strawn DG, Scheckel KG, Sparks DL, Grundl TJ (1998). Kinetics and mechanisms of metal sorption at the mineral-water interface. Mineral-water interfacial reactions: kinetics and mechanisms.

[CR47] Stevenson FJ (1994) Humus chemistry: genesis, composition, reactions. John Wiley and Sons Inc.

[CR48] Thomas B (2000) Solid phase extraction for the removal of organic-iron complexes in contaminated groundwater. MSc thesis, The University of Leeds

[CR49] Weber F-A (2009). Multi-metal contaminant dymanics in temporarily flooded soil under sulfate limitation. Geochim Cosmochim Acta.

[CR50] Wessa P (2014) Free Statistics Software, Office for Research Development and Education, Version 1.1.23-r7. [Online]. [Accessed 24th September 2014]. Available from: http://www.wessa.net

[CR51] Wu J (2001). Copper(II) humate mobility in kaolinite soil. Eng Geol.

[CR52] Wu J (2002). Effect of humic substances on Cu(II) solubility in kaolin-sand soil. J Hazard Mater.

[CR53] Yin YJ (2002). The importance of oarganic matter distribution and extract soil: solution ratio on the desorption of heavy metals from soils. Sci Total Environ.

