# Supplementary material for: Leaching of copper and nickel in soil-water systems contaminated by bauxite residue (red mud) from Ajka, Hungary: the importance of soil organic matter
Source: Environ Sci Pollut Res Int. 2015 Mar 12;22(14):10800–10. doi: 10.1007/s11356-015-4282-4 (PMC4490175; doi:10.1007/s11356-015-4282-4)
Supplement: Supplementary file 1 — (DOCX 707 kb) [file 11356_2015_4282_MOESM1_ESM.docx]

**Online Resource for:**

**Leaching of copper and nickel in soil-water systems contaminated by bauxite residue (red mud) from Ajka, Hungary: The importance of organic matter**

**Cindy L. Lockwood^1#^, Douglas I. Stewart^2^, Robert J. G. Mortimer^1$^, William M. Mayes^3^, Adam P. Jarvis^4^, Katalin Gruiz^5^ and Ian T. Burke^1^***

^1^School of Earth and Environment, University of Leeds, Leeds, LS2 9JT, UK. ^*^Corresponding Author’s E-mail: [I.T.Burke@leeds.ac.uk](mailto:I.T.Burke@leeds.ac.uk); Phone: +44 113 3437532; Fax: +44 113 3435259

^2^School of Civil Engineering, University of Leeds, Leeds, LS2 9JT, UK.

^3^Centre for Environmental and Marine Sciences, University of Hull, Scarborough, YO11 3AZ, UK

^4^School of Civil Engineering and Geosciences, Devonshire Building, Newcastle University, Newcastle Upon Tyne, NE1 7RU, UK

^5^Department of Applied Biotechnology and Food Science, Budapest University of Technology and Economics, 1111 Budapest, St Gellért sq. 4, Hungary

^#^Present address: Eberhard-Karls-University Tübingen, Centre for Applied Geoscience, 72076 Tübingen, Germany

^$^Present address: School of Animal, Rural and Environmental Sciences, Nottingham Trent University Brackenhurst campus, Southwell, Nottinghamshire, NG25 0QF

Prepared for Environmental science and Pollution Research, 11 February 2015

This section consists of 7 pages and 2 Tables and 4 Figures

**Table A. Full XRF analysis of red mud and soils**

Concentrations of selected elements present in the red mud sample and soil samples prior to amendment ([Lehoux et al. 2013](#_ENREF_1))

| **Major Elements**  (Weight %Oxides) | **Red Mud** | **Organic-rich soil** | **Wetland soil** | **Sandy soil** |
| --- | --- | --- | --- | --- |
| SiO_2_ | 12.9 | 72.9 | 80.6 | 89.8 |
| TiO_2_ | 5.24 | 0.48 | 0.33 | 0.37 |
| Al_2_O_3_ | 16.0 | 8.9 | 6.4 | 4.2 |
| Fe_2_O_3_ | 38.5 | 3.0 | 1.8 | 1.7 |
| MnO | 0.31 | 0.04 | 0.03 | 0.06 |
| MgO | 0.61 | 0.84 | 0.98 | 0.06 |
| CaO | 8.0 | 1.0 | 2.27 | 0.60 |
| Na_2_O | 8.0 | 1.0 | 0.90 | 0.71 |
| K_2_O | 0.10 | 1.6 | 1.3 | 0.87 |
| P_2_O_5_ | 0.19 | 0.11 | 0.07 | 0.08 |
| SO_3_ | 0.36 | 0.02 | 0.02 | 0.006 |
| *LOI* | *8.0* | *10.2* | *5.1* | *1.8* |
| **Minor Elements** (mg kg^-1^) |  |  |  |  |
| As | 196 | 11 | 8 | 2 |
| Ba | 66 | 396 | 267 | 148 |
| Ce | 607 | 47 | 34 | 17 |
| Co | 59 | 11 | 5 | 3 |
| Cr | 864 | 68 | 62 | 50 |
| Cu | 104 | 12 | 6 | 2 |
| Ga | 26 | 10 | 6 | 4 |
| La | 283 | 26 | 18 | 10 |
| Mo | 15 | 1 | 1 | 1 |
| Ni | 361 | 23 | 14 | 5 |
| Pb | 215 | 25 | 12 | 9 |
| Sb | 22 | 1 | 2 | 1 |
| Sr | 318 | 78 | 94 | 47 |
| Th | 98 | 6 | 4 | 2 |
| U | 21 | 3 | 2 | 1 |
| V | 1132 | 72 | 51 | 30 |
| W | 17 | <1 | <1 | <1 |
| Zn | 162 | 52 | 26 | 21 |
| Zr | 1223 | 122 | 102 | 88 |

**Table B. Sequential extraction data for Cu and Ni.**

Amount of Cu and Ni Leached from triplicate Ajka red mud samples collected from location K1 in [Mayes et al. (2011)](#_ENREF_3) during sequential extractions ([Rauret et al. 1989](#_ENREF_4)). Values in parenthesis are percentages of total Cu or Ni leached.

| Leaching Step | Cu (mg kg^-1^) | Ni (mg kg^-1^) |
| --- | --- | --- |
| 1 mol L^-1^ Mg(Cl)_2_ at pH 7 | 0.1 ±0.0 (0.2 ±0.0%) | 0.1 ±0.0 (0.2 ±0.0%) |
| 1 mol L^-1^ NaOAc / HOAc at pH 5 | 24.9 ±1.8 (38 ±3%) | 2.7 ±0.1 (0.7 ±0.0%) |
| 0.5 mol L^-1^ NH_2_OH.HCl / HCl at pH 1.5 | 18.5 ±1.0 (28 ±2%) | 54.4 ±3.2 (14 ±1%) |
| 30% H_2_O_2_ / HNO_3_ at pH 2 | 8.8 ±3.0 (13 ±5%) | 43.3 ±0.6 (11 ±0.2%) |
| Concentrated HF / HCl / HNO_3_ | 13.4 ±1.1 (20 ±2%) | 280.7 ±2.7 (74 ±0.7%) |


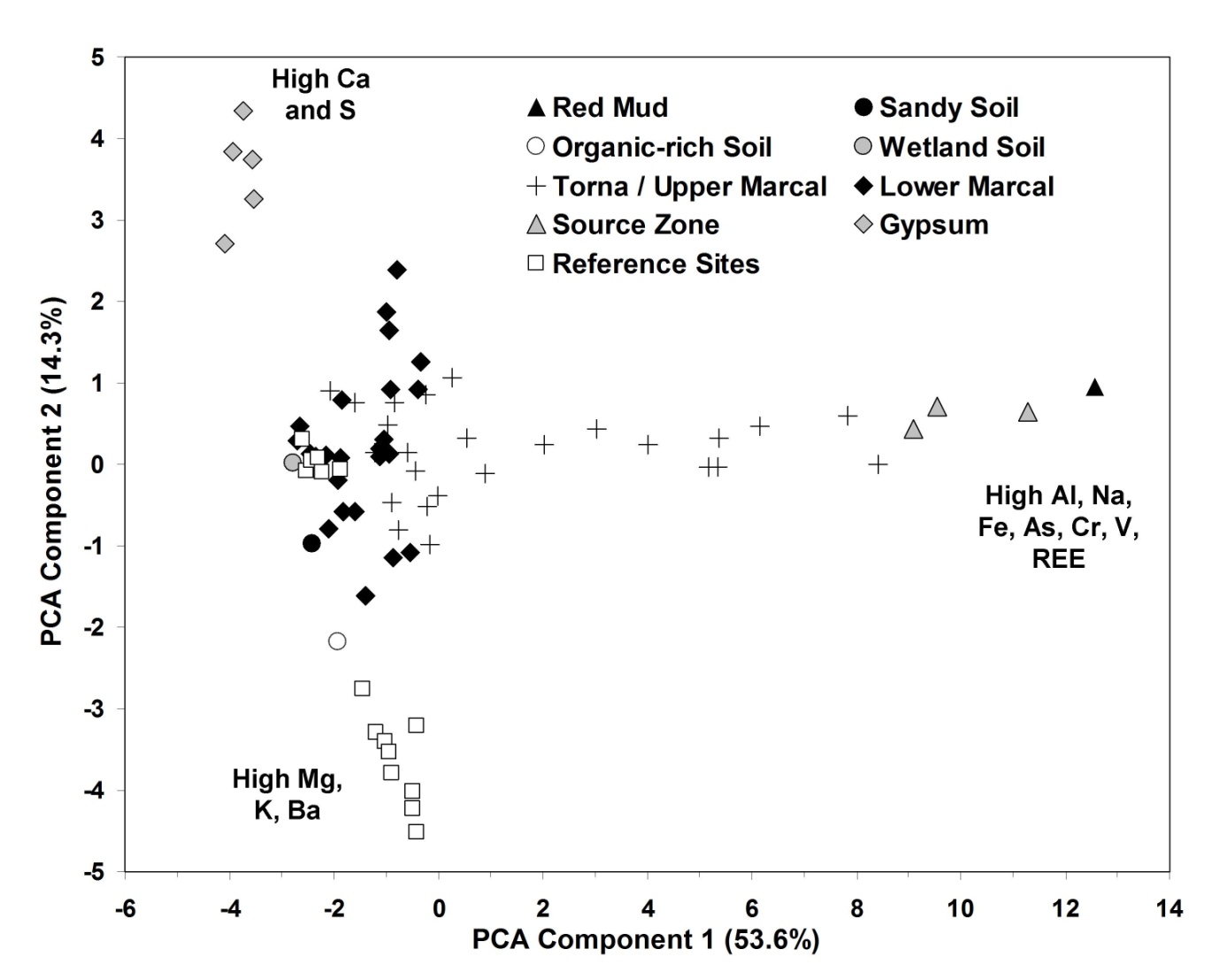


**Figure A.** Taken from [Lehoux et al. (2013)](#_ENREF_1) **-**  Principle Component Analysis - shows the samples used in this study in the broader context of large scale soil and sediment sampling efforts across a 100km reach of the Torna-Marcal-Raba River system in the immediate aftermath of the spill ([Mayes et al. 2011](#_ENREF_3)). Each point represents a single sample station and three end members are apparent in the PCA. Samples of red mud from the Ajka repository plot at the extreme right hand side of the figure, being characterised by enriched Fe, Al, Ti, Na, Cr, V and various other trace elements. The red mud samples used in this study (labelled ‘Red Mud’ in the legend) plot alongside those red mud taken from previous sampling efforts at the same site (labelled ‘Source’, sample K1 from [Mayes et al. (2011)](#_ENREF_3). To the upper left of Figure A, samples enriched with Ca and S are indicative of river sediments in reaches subject to extensive gypsum dosing (to neutralise the high pH) shortly after the spill. To the lower left, unaffected reference sites are apparent as a final end member and are relatively enriched in K, Ba and Mg, consistent with bedrock lithology ([Mayes et al. 2011](#_ENREF_3)). The samples plotting between these end members represent mixing of these different extremes, for example the samples that plot in a roughly straight horizontal line from the red mud to the left hand side of Figure A represent dilution of red mud through mixing with soils and sediments with distance downstream from the source area. The soil samples used in this study all plot in a group on the left hand side with unaffected sites from the lower Marcal River and reference samples showing them to be representative of the broad geochemical constituent of unaffected sediments in the Marcal catchment. The pH of the waters in this area are neutral (pH 7 – 8) as this area is dominated by dolomite and limestone bedrock ([Mayes et al. 2011](#_ENREF_3)).

**
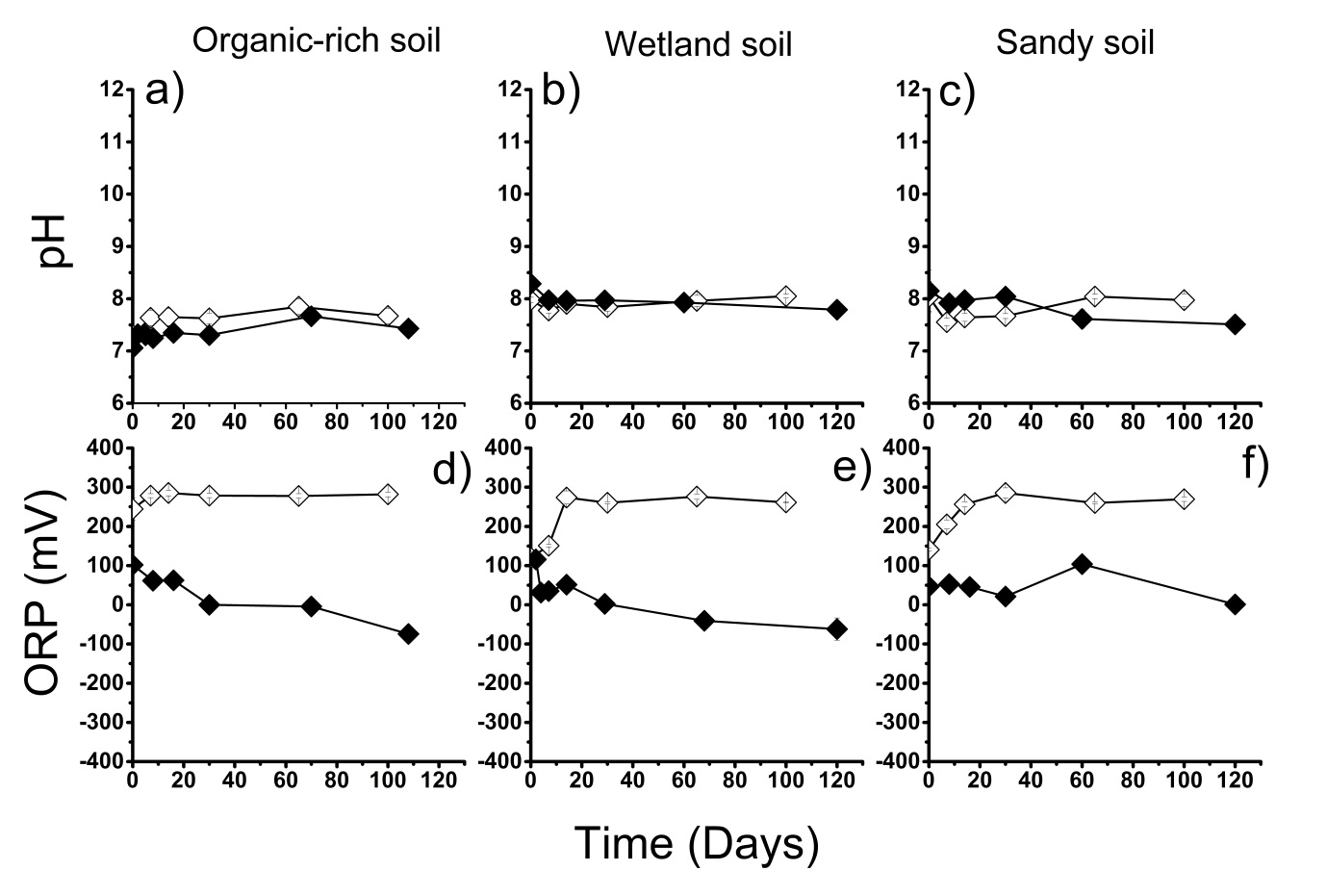
**

**Figure B –** pH and ORP (as an indicator for Eh) in unamended control experiments over time. Full symbols = anaerobic experiments, empty symbols = aerobic experiments. Error bars are 1 σ of triplicate results (where not shown, errors are within the symbol size).

**
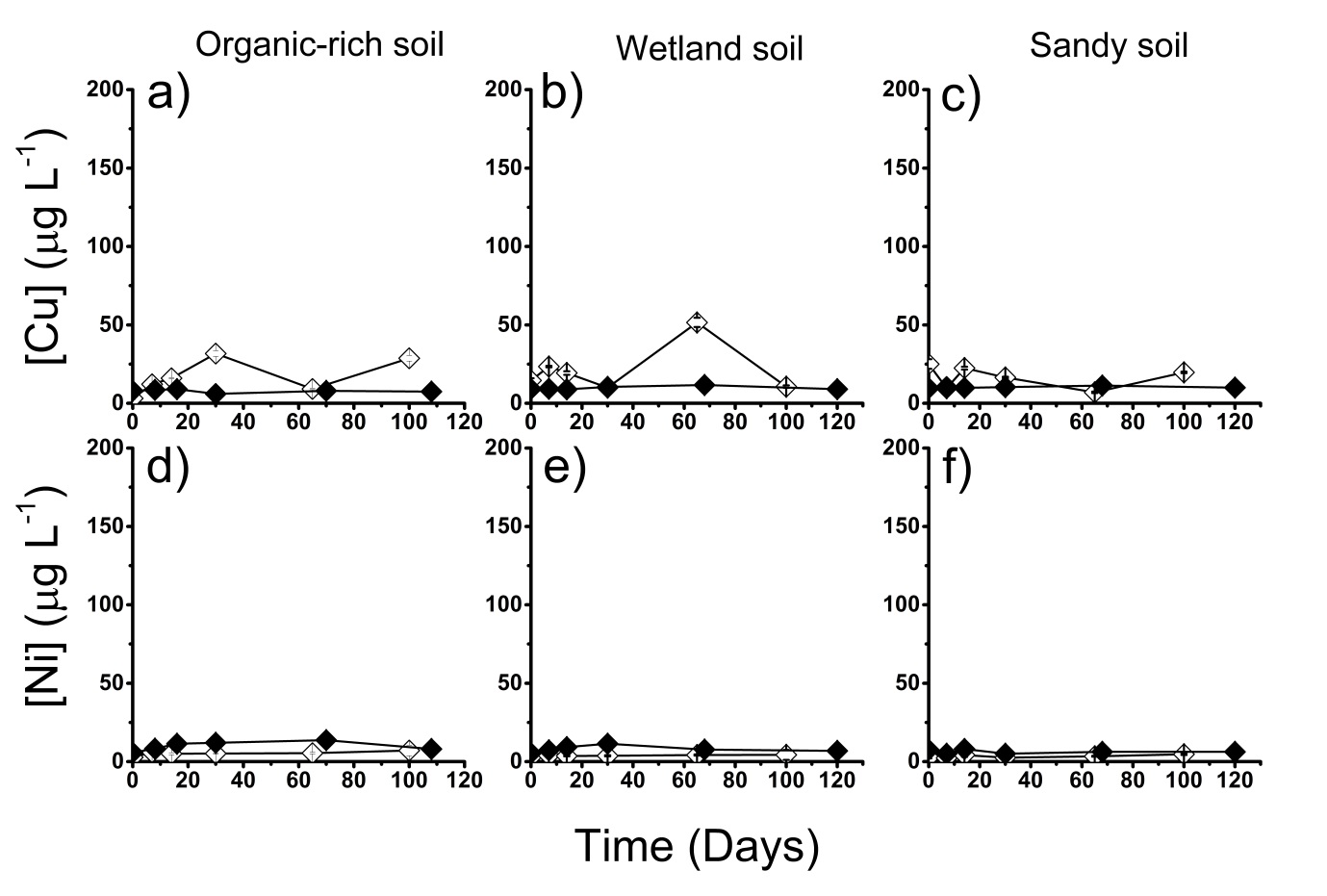
**

**Figure C –** Evolution of aqueous Cu and Ni over time in unamended control experiments. Full symbols = anaerobic, empty symbols = aerobic. Error bars are 1 σ of triplicate results (where not shown, errors are within the symbol size).

**
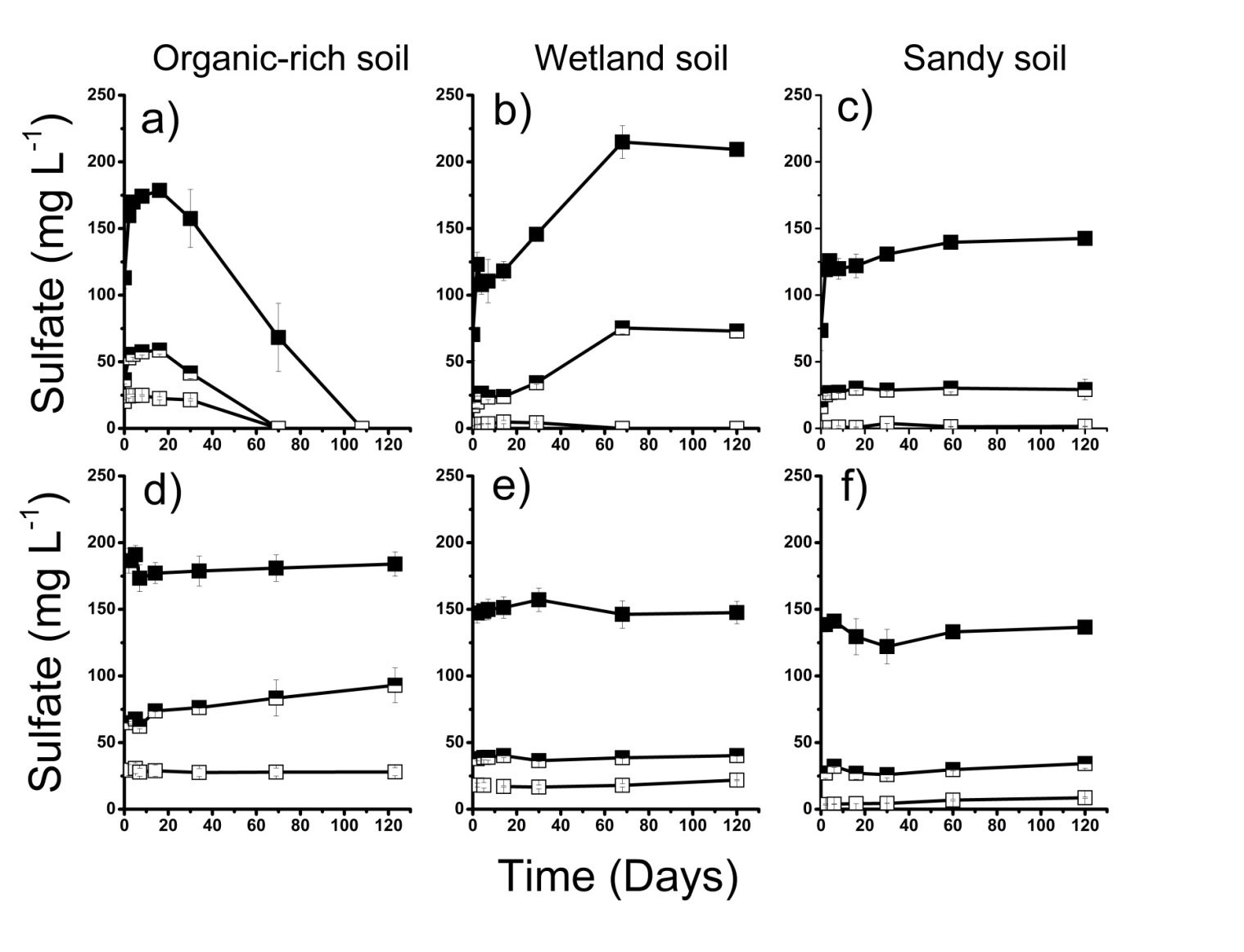
**

**Figure D –** Evolution of sulfate over time in anaerobic red mud amended soil experiments (a-c), and the associated controls (d-f). Full symbols = 33% RM, half squares = 9% RM and empty squares = no RM. Error bars are 1 σ of triplicate results (where not shown, errors are within the symbol size). Sulfate was not recorded for aerobic experiments as sulfate reducing conditions were not anticipated. See [Lockwood et al. (2014)](#_ENREF_2).

References

Lehoux AP, Lockwood CL, Mayes WM, Stewart DI, Mortimer RJG, Gruiz K, Burke IT (2013): Gypsum addition to soils contaminated by red mud: implications for aluminium, arsenic, molybdenum and vanadium solubility. Environmental Geochemistry and Health 35, 643-656

Lockwood CL, Mortimer RJG, Stewart DI, Mayes WM, Peacock CL, Polya DA, Lythgoe PR, LeHoux AP, Gruiz K, Burke IT (2014): Mobilisation of arsenic from bauxite residue (red mud) affected soils: Effect of pH and redox conditions. Appl. Geochem. 51, 268-277

Mayes WM, Jarvis AP, Burke IT, Walton M, Feigl V, Klebercz O, Gruiz K (2011): Dispersal and Attenuation of Trace Contaminants Downstream of the Ajka Bauxite Residue (Red Mud) Depository Failure, Hungary. Environmental Science & Technology 45, 5147-5155

Rauret G, Rubio R, Lopezsanchez JF (1989): OPTIMIZATION OF TESSIER PROCEDURE FOR METAL SOLID SPECIATION IN RIVER SEDIMENTS. International Journal of Environmental Analytical Chemistry 36, 69-83
